# Early Prediction of Acute Kidney Injury Following Liver Transplantation: Development and Validation of a Clinical Risk Model

**DOI:** 10.1016/j.jceh.2025.103179

**Published:** 2025-08-29

**Authors:** Yuzhi Wei, Ziheng Qi, Wenyan Wu, Chunyu Feng, Bo Yang, Haolin Yin, Caiyun Zhang, Xiaoyan Gao, Haotian Wu, Shichao Sun, Wenfang Zhang, Huan Zhang

**Affiliations:** ∗ Department of Anesthesiology, Beijing Tsinghua Changgung Hospital, School of Clinical Medicine, Tsinghua Medicine, Tsinghua University, Beijing 102218, China; † Department of Anesthesiology, Dongying People's Hospital of Shandong Provincial Hospital Group, Dongying 257091, China; ‡ Department of Anesthesiology, Central People's Hospital of Zhanjiang, Zhanjiang 524045, China; § Department of Cardiology, Yantai Affiliated Hospital of Binzhou Medical University, Yantai 264100, China

**Keywords:** liver transplantation, acute kidney injury, prediction model, validation

## Abstract

**Background:**

The first 48 h following liver transplantation (LT) represent a critical therapeutic window. Early identification of patients who are at high risk of developing acute kidney injury (AKI) can optimize treatment strategies and improve patient outcomes. This study aimed to develop and validate a clinical risk prediction model for AKI within 48 h following LT by utilizing preoperative and intraoperative parameters.

**Methods:**

A total of 453 adult LT recipients treated at the Beijing Tsinghua Changgung Hospital between January 2018 and October 2022 were enrolled. Patients were randomly assigned to a development cohort and a validation cohort at a 6:4 ratio. AKI was diagnosed using the 2012 Kidney Disease Improving Global Outcomes (KDIGO) criteria. Univariate and multivariate logistic regression analyses identified clinical factors associated with early AKI. A predictive model was constructed and internally validated. Additionally, stages 2 and 3 AKI, as defined by the KDIGO criteria, were classified as severe AKI. Independent risk factors for severe AKI within 48 h following LT were similarly identified using logistic regression analyses.

**Results:**

At 48 h following LT, 125 (46%) patients developed AKI. Univariate analysis identified 17 potential predictive factors for AKI, including preoperative hepatic encephalopathy (HE), a history of alcohol-associated cirrhosis, body mass index ≥28 kg/m^2^, and a prognostic nutritional index > 43 (*P* < 0.1). A backward stepwise regression model was utilized to develop a clinical risk prediction model incorporating the following variables: HE, alcohol-associated cirrhosis, preoperative albumin–bilirubin score ≥ −1.78, operation time ≥560 min, and intraoperative fresh frozen plasma transfusion volume (per 1000 mL). The model achieved an area under the curve (AUC) of 0.760 (*P* < 0.05) in the development cohort and 0.759 (*P* < 0.05) in the validation cohort. The calibration curve indicated excellent agreement between predicted and observed probabilities of early AKI (*P* > 0.05). Multivariate logistic regression analysis identified the preoperative model of end-stage liver disease score ≥14, operation time ≥560 min, intraoperative blood loss ≥1000 mL, intraoperative urine output <1000 mL, and elevated lactic acid level as independent risk factors for severe AKI.

**Conclusion:**

The proposed predictive model could promote the identification of high-risk LT recipients immediately following surgery, enabling clinicians to intervene early to mitigate the risk of developing AKI within 48 h postoperatively. This approach has the potential to improve patient prognosis by supporting timely and targeted management strategies.

## Graphical abstract

**Figure:**
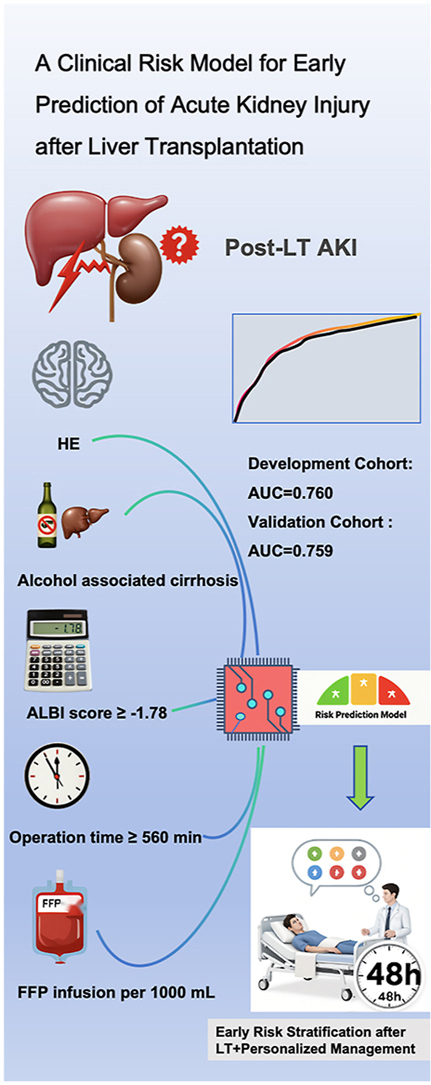

Since the first liver transplantation (LT) performed by Starzl *et al.* in 1963,^1^ the procedure has exhibited a fundamental role in the treatment of end-stage liver disease. However, despite significant advancements, complications such as bleeding, rejection, bile leakage, portal vein thrombosis, and acute kidney injury (AKI) continue to adversely impact patient outcomes.^1^, ^2^, ^3^, ^4^ The incidence of AKI following LT ranges from 12.7% to 64.1%,^5^, ^6^, ^7^ and studies have demonstrated its association with increased postoperative infection risk, prolonged hospital stay, elevated short- and long-term mortality rates, and augmented healthcare costs.^8^^,^^9^ Furthermore, AKI may deteriorate into renal failure requiring dialysis, with some cases advancing to chronic kidney disease (CKD) or end-stage renal disease.^10^ Consequently, close monitoring and proactive measures to prevent AKI following LT are critically important.

Diagnosing AKI following LT is complex due to its multifactorial origins and potential for occult onset, complicating predictive efforts. The major risk factors include hepatorenal syndrome,^11^ a history of hypertension,^12^ diabetes,^13^ body mass index (BMI),^14^ ischemia-reperfusion injury (IRI), intraoperative hypotension, surgical duration,^15^ postoperative immunosuppressants, and infections.^16^ Advancements in organ preservation techniques and surgical innovations have significantly altered the risk profile for post-LT AKI. Timely identification of these evolving risk factors is essential to optimize patient outcomes.

The incidence of AKI following LT significantly varies, which could be attributed to inconsistent diagnostic criteria across studies. Early predictive models have mainly concentrated on severe AKI requiring renal replacement therapy (RRT).^17^ However, growing recognition of the clinical significance of mild AKI has prompted a transition toward utilizing the acute kidney injury network and the KDIGO criteria.^18^ Approximately 80% of post-LT AKI cases occur within the first 48 h and are primarily associated with preoperative and intraoperative risk factors.^19^ This timeframe is critical to determine treatment efficacy, highlighting the importance of identifying high-risk patients for tailored immunosuppressive therapy and renal perfusion.

Recent studies using the 2012 KDIGO criteria have examined AKI primarily within the first postoperative week,^12^ while risk factors could significantly vary between the first 48 h and the first week owing to infections and nephrotoxic medications. For instance, Schiefer *et al.*’s predictive model, based on the KDIGO and a 48-h endpoint, was limited by a small sample size.^20^ In Birhara *et al.*’s research, mild AKI cases were excluded on the basis of the International Club of Ascites criteria as they concentrated solely on identifying stages 2 and 3.^21^ Consequently, the risk factors for AKI within the first 48 h following LT remain elusive and warrant further investigation.

New indicators, such as the prognostic nutritional index,^22^ have recently exhibited promise, while they are rarely integrated into AKI predictive models. The present study employed the KDIGO criteria, emphasizing the critical first 48 h postoperatively, and incorporated novel predictive markers to improve the precision and clinical utility of AKI prediction. The findings may assist clinicians with an effective tool for the early identification of high-risk patients, enabling timely interventions, reducing postoperative complications, and enhancing patient outcomes.

## METHODS

The study was approved by the Institutional Review Board of the Beijing Tsinghua Changgung Hospital, School of Clinical Medicine, Tsinghua University (China; Approval No. 22216-4-01), and the requirement for written informed consent was waived. The research was conducted in accordance with the Transparent Reporting of Individual Prognosis or Diagnosis (TRIPOD) guidelines.

### Setting and Data Sources

A single-center retrospective study was conducted at the Beijing Tsinghua Changgung Hospital affiliated to Tsinghua University, which is a tertiary teaching institution with expertise in organ transplantation and anesthesia. Clinical and laboratory data from consecutive liver transplant patients over a nearly five-year period were extracted from the electronic medical record system, ensuring accuracy through a dual verification. Preoperative, intraoperative, and postoperative data were collected. Preoperative and intraoperative variables were utilized to develop and validate the AKI prediction model, while postoperative data were utilized to define endpoints and outcomes.

### Study Population

The study included all consecutive patients who underwent LT at the Beijing Tsinghua Changgung Hospital affiliated to Tsinghua University from January 1, 2018, to October 15, 2022. All recipients demonstrated pretransplant control of primary etiologies: hepatitis B virus (HBV) DNA <20 IU/mL (institutional threshold), hepatitis C virus (HCV) sustained virological response after direct-acting antiviral therapy, or ≥6 months of documented alcohol abstinence for alcohol-related liver disease. Exclusion criteria were as follows: (1) patients who aged under 18 years, (2) those with a history of post-renal transplantation, (3) cases involving LT combined with other organ transplants, (4) preoperative dependence on RRT, (5) cases undergoing secondary or subsequent LT, (6) patients with preexisting CKD, defined as the baseline estimated glomerular filtration rate (eGFR) < 60 mL/min/1.73 m^2^, were excluded. Notably, acute kidney injury (AKI) not requiring RRT was not an exclusion criterion, provided baseline eGFR met the threshold and (7) patients with severe illness led to surgical termination.

### Anesthesia Procedure and monitoring

The procedures were carried out by a specialized team following a standardized protocol. Venous access was established, and monitoring included electrocardiogram, oxygen saturation (SpO_2_), blood pressure, heart rate, arterial blood pressure, and central venous pressure, with additional parameters, such as cardiac output and stroke volume variation as needed. Anesthesia induction involved midazolam (0.03–0.05 mg/kg), etomidate (0.2–0.3 mg/kg), sufentanil (0.3–0.5 μg/kg), and cisatracurium (0.2 mg/kg). Following intubation, mechanical ventilation was set with a tidal volume of 8–10 mL/kg, respiratory rate of 12–15/min, and partial end-tidal carbon dioxide of 35–45 mmHg. Anesthesia maintenance consisted of propofol (2–8 mg/kg/h) or dexmedetomidine (0.2–0.4 μg/kg/h), sufentanil (0.15–0.7 μg/kg/h), cisatracurium (0.06–0.18 mg/kg/h), and sevoflurane (1–2 vol%), targeting a bispectral index of 40–60. Circulatory stability was maintained through fluids, vasopressors, or adjustments in anesthesia depth. Regarding fluid and postoperative management, crystalloids (Plasma-Lyte), colloids (5% albumin), packed red blood cells (PRBCs), and fresh frozen plasma (FFP) were administered based on bleeding, oozing, and mean arterial pressure (MAP). Postoperatively, patients were transferred to the intensive care unit with analgesia pumps for ongoing care.

### Liver Transplantation

This study adhered to the ethical guidelines of the 1975 Declaration of Helsinki, regulations of the Organ Transplantation Committee, National guidelines for donation after cardiac death in China,^23^ and current Chinese laws. Each transplant was approved by the Ethics Committee of the Beijing Tsinghua Changgung Hospital, School of Clinical Medicine, Tsinghua University. All organ donors were controlled donors following cardiac death.^24^ Donor organs were allocated transparently through the China Organ Transplant Response System (https://www.cot.org.cn/). No organs from executed prisoners were used.

All liver transplants were performed by an experienced surgical team at the Beijing Tsinghua Changgung Hospital, School of Clinical Medicine, Tsinghua University, utilizing either the “classic” liver transplant or the “piggyback” technique.

### Postoperative Immunosuppressive Therapy

This study developed a risk-stratified immunosuppression protocol based on multidimensional preoperative assessments. For recipients with renal insufficiency, heightened rejection risk from prior immunosuppressive exposure, or significant infection risks, a two-phase induction regimen combining basiliximab with corticosteroids was implemented: 500 mg methylprednisolone and 20 mg basiliximab were administered intravenously prior to graft reperfusion, followed by a second basiliximab dose (20 mg IV) on postoperative day 4. Recipients with normal renal function and no signs of infection were initiated on standard tacrolimus therapy (1.5–2.0 mg twice daily) within 24 h posttransplant. Those developing dynamic renal impairment (defined as a sustained upward creatinine trend or exceeding the upper normal limit) received delayed tacrolimus initiation at 72 h postoperatively with a reduced-dose regimen (1 mg twice daily). All patients underwent tacrolimus trough concentration monitoring (target range: 8–12 ng/mL) starting 24 h after the initial dose, with real-time dose adjustments guided by renal function indices and pharmacokinetic data. This protocol achieved precision through risk stratification: high-risk patients received intensified basiliximab induction to delay calcineurin inhibitor exposure and mitigate nephrotoxicity, while low-risk patients benefited from early tacrolimus initiation to ensure adequate immunosuppression. Therapeutic drug monitoring further enabled individualized dosing optimization. The stratified approach effectively balanced graft protection, rejection prevention, and postoperative complication management, addressing critical clinical priorities in liver transplantation.

### Candidate Predictors

This study identified preoperative and intraoperative predictors of AKI after LT based on a literature review.^22^^,^^25^ Newly proposed preoperative predictors, such as the albumin–bilirubin (ALBI) score and the prognostic nutritional index (PNI), were included. A total of 27 candidate predictors were analyzed, comprising 17 preoperative and 10 intraoperative factors (Table 1). Preoperative factors included baseline characteristics (e.g., age, gender, and BMI), comorbidities (diabetes and hypertension), liver disease complication (hepatic encephalopathy), severity scores (the model of end-stage liver disease (MELD) score and the Child–Pugh score), nutritional and liver function indicators (PNI and ALBI), laboratory results (serum creatinine and serum urea nitrogen), and pathogenesis of liver diseases (e.g., HBV , HCV, alcohol-associated cirrhosis, hepatocellular carcinoma and primary biliary cirrhosis). Intraoperative factors included hemodynamics, surgical parameters (operation time, anhepatic phase, cold ischemia duration), maximum lactate, urine output, blood loss, and transfusions (5% albumin, PRBCs, and fresh frozen plasma (FFP)). Definitions that were described are as follows:

**Table 1:** Descriptions of Preoperative and Intraoperative Predictors for Development Cohort.

Predictors	Total (n = 272)	Patients with AKI at 48h following LT (n = 125)	Patients without AKI at 48h following LT (n = 147)	*P*
Preoperative predictors				
Age, Median (IQR), years	52.0 (44.0,60.0)	52.0 (43.0,58.0)	53.0 (45.5, 60.5)	0.148
Female, n (%)	59 (21.7)	29 (23.2)	30 (20.4)	0.578
BMI, Median (IQR), kg/m^2^	23.7 (21.6, 26.2)	24.1 (22.0, 27.3)	23.3 (21.3, 25.6)	0.025
BMI ≥28 kg/m^2^, n (%)	45 (16.5)	28 (22.4)	17 (11.6)	0.017
MELD score, Median (IQR)	14.0 (9.0, 22.0)	17.0 (12.0, 25.0)	12.0 (8.0, 18.0)	< 0.001
MELD score ≥14, n (%)	140 (51.5)	83 (66.4)	57 (38.8)	< 0.001
Child–Pugh score, Median (IQR)	8.0 (6.0, 10.0)	9.0 (7.0, 11.0)	8.0 (6.0, 10.0)	< 0.001
Child–Pugh score ≥7, n (%)	192 (70.6)	102 (81.6)	90 (61.2)	< 0.001
ALBI score, Median (IQR)	−1.7 (−2.3, −1.3)	−1.5 (−1.9, −1.2)	−1.9 (−2.4, −1.4)	< 0.001
ALBI score ≥ −1.78, n (%)	150 (55.1)	86 (68.8)	64 (43.5)	< 0.001
PNI, Median (IQR)	38.2 (35.0, 42.2)	37.5 (34.6, 40.8)	38.7 (35.8, 43.7)	0.028
PNI <43, n (%)	211 (77.6)	105 (84)	106 (72.1)	0.019
Diabetes mellitus, n (%)	59 (21.7)	28 (22.4)	31 (21.1)	0.794
Hypertension, n (%)	48 (17.6)	17 (13.6)	31 (21.1)	0.106
HE, n (%)	38 (14.0)	27 (21.6)	11 (7.5)	< 0.001
Pathogenesis of liver disease, n (%)				
HBV hepatitis	174 (64.0)	75 (60)	99 (67.3)	0.208
HCV hepatitis	11 (4.0)	5 (4)	6 (4.1)	0.973
Hepatocellular carcinoma	57 (21.0)	20 (16)	37 (25.2)	0.066
Alcohol-related cirrhosis	33 (12.1)	22 (17.6)	11 (7.5)	0.011
Primary biliary cirrhosis	10 (3.7)	4 (3.2)	6 (4.1)	0.758
Pre-transplant BUN, Median (IQR),mmol/L	5.2 (3.9, 7.1)	5.3 (4.0, 7.5)	5.1 (3.7, 6.8)	0.158
Pre-transplant SCr, Median (IQR),mmol/L	59.3 (49.9, 76.2)	59.0 (49.0, 78.5)	60.0 (51.0, 75.8)	0.658
Intraoperative predictors				
Duration of hypotension ≥20 min, n (%)	139 (51.1)	74 (59.2)	65 (44.2)	0.014
Operation time ≥560 min, n (%)	122 (44.9)	77 (61.6)	45 (30.6)	< 0.001
Cold ischemia time ≥400 min, n (%)	86 (31.6)	50 (40)	36 (24.5)	0.006
Anhepatic phase ≥60 min, n (%)	169 (62.1)	87 (69.6)	82 (55.8)	0.019
5% Albumin infusion ≥2000 mL, n (%)	160 (58.8)	86 (68.8)	74 (50.3)	0.002
PRBC_S_ infusion, Median (IQR), units	4.0 (0.0, 8.0)	4.0 (0.0, 8.0)	2.0 (0.0, 4.0)	< 0.001
FFP Infusion, Median (IQR), ml	400 (0, 400)	400 (0, 800)	0 (0, 400)	< 0.001
Blood loss ≥1000 mL, n (%)	65 (23.9)	43 (34.4)	22 (15)	< 0.001
Urine output <1000 mL, n (%)	83 (30.5)	37 (25.2)	46 (36.8)	0.038
Serum lactic acid maximum, Median (IQR), mmol/L	3.6 (2.7, 4.6)	4.0 (3.0, 5.2)	3.3 (2.5, 4.0)	< 0.001

Abbreviations: ALBI, albumin–bilirubin; BMI, body mass index; BUN, blood urea nitrogen; FFP, fresh frozen plasma; HE, hepatic encephalopathy; LT, liver transplantation; MELD, model for end-stage liver disease; PNI, prognostic nutritional index; PRBCs, packed red blood cells; SCr, Serum Creatinine.

AKI: AKI was diagnosed according to the 2012 KDIGO criteria^26^ (Supplementary Table 1): serum creatinine rise ≥50% or ≥ 26.5 μmol/L within 48 h following surgery. Baseline creatinine was the most recent preoperative value, and kidney function was assessed using eGFR.

Severe AKI: Defined as stage 2 or 3 AKI in accordance with the KDIGO criteria.^26^

Hypotension: Defined as intraoperative MAP < 60 mmHg or systolic blood pressure (SBP) < 90 mmHg.

Severe hypotension: Defined as intraoperative MAP <60 mmHg sustained for >15 consecutive minutes.

Duration of hypotension: Refers to the cumulative intraoperative time (minutes) during which either: MAP <60 mmHg, or SBP <90 mmHg.

MELD score = 3.78 × ln (total bilirubin (μmol/L)) + 11.2 × ln (international normalized ratio) +9.57 × ln (serum creatinine (μmol/L)) +6.43 × D (where (D = 0) for biliary or alcoholic liver disease, and (D = 1) for other etiologies).^27^

ALBI score = log_10_ bilirubin level (μmol/L) × 0.66+ albumin level (g/L) × (−0.085).^28^

PNI = albumin level (g/L) + 5 × lymphocyte count ( × 10^9^/L).^22^

The diagnosis of hepatic encephalopathy (HE) was made in accordance with the standardized West Haven criteria as documented in the patient’s admission records.

### Statistical Analysis

This retrospective study did not include formal sample size calculations, and the sample size was determined based on the available data. The cohort was randomly categorized into two datasets: 60% (272 patients) for model development and 40% (181 patients) for internal validation. Descriptive statistics were computed, and continuous variables were assessed for normality. Normally distributed variables were presented as mean ± standard deviation (x ± s) and compared using the independent-samples t-test. Abnormally distributed variables were expressed as median (M) with interquartile range) and compared using the Mann–Whitney U test. Categorical variables were presented as count and percentage, and comparisons were conducted using Chi-square test or the Fisher’s exact test.

### Model Development

Potential predictors were selected based on clinical experience, pathophysiology, the existing literature, and their relevance to postoperative AKI. Continuous variables were dichotomized using cut-off values derived from receiver operating characteristic (ROC) curves, with the objective of optimizing Youden’s index. The established clinical thresholds (BMI ≥28 kg/m^2^; blood loss ≥1000 mL) were maintained, while the statistically optimized values underwent integer rounding in the context of clinical application. This is illustrated by the reduction of Child–Pugh score from 7.5 to 7, aligning with the criteria for Class B cirrhosis (7–9 points) for immediate prognostic stratification. Full derivation metrics can be found in the supplementary material (Supplementary Table 2). Univariate analysis was undertaken to identify variables significantly associated with AKI (*P* < 0.05). Variables with *P* < 0.1 were involved in the multivariate logistic regression analysis. Collinearity was assessed using the variance inflation factor (VIF), and variables with a VIF >5 were excluded. Stepwise backward logistic regression was employed to select predictors, optimizing the model by retaining the fewest predictors necessary to maintain performance. The final model was developed to predict the risk of AKI within 48 h following LT.

### Model Validation

The model’s discrimination and calibration were evaluated using the validation dataset. Discrimination, the model’s ability to differentiate among outcomes (e.g., AKI occurrence), was assessed using area under ROC (AUROC) and its 95% confidence interval (CI). An AUROC of 0.5 indicates poor discrimination, while 1.0 indicates perfect performance. Calibration, reflecting consistency between predicted and actual risks, was tested using the Hosmer–Lemeshow test, Loess-smoothed calibration plots, and calibration slopes.

### Risk Factor Analysis for Severe AKI Within 48 h Following LT

Risk factors for severe AKI within 48 h following LT were analyzed. Significant predictors (*P* < 0.05) identified using the univariate analysis were included in the multivariate logistic regression analysis.

All *P* values were two-tailed, and a *P* value <0.05 was considered statistically significant. Statistical analysis was carried out by using SPSS 27.0 (IBM, Armonk, NY, USA) and R 4.4.2 (https://www.r-project.org/) software.

## RESULTS

### Baseline Characteristics

**Figure 1:**
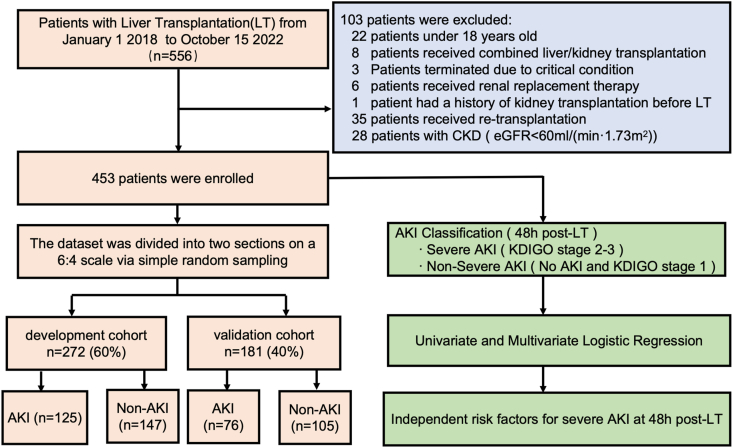
Flowchart of patient selection, cohort division, and analysis of independent risk factors for severe AKI. AKI, acute kidney injury; eGFR: estimated glomerular filtration rate; KDIGO, kidney disease improving global outcomes; LT, liver transplantation.

This study included 453 patients who underwent LT after excluding 122 cases based on the predefined criteria (Figure 1). AKI within 48 h posttransplantation was assessed using the 2012 KDIGO criteria (Supplementary Table 1). Among them, 272 patients were assigned to the development cohort and 181 to the validation cohort. There were no significant differences in the incidence of AKI (46% vs. 42%, *P* = 0.405) or baseline characteristics between the two cohorts (Supplementary Table 3). In the development cohort, the median age was 52 years (range, 41–63 years), and 78.3% were male. End-stage liver disease etiologies included hepatocellular carcinoma (16.0%), predominantly HBV hepatitis (64%) (Table 1).

Our analysis of clinical data indicated that 201 of the 453 patients (44.4%) developed AKI within 48 h after surgery. Based on the KDIGO criteria, the cases were categorized as Stage 1 (n = 126), Stage 2 (n = 41), or Stage 3 (n = 34). Within the first 48 h postoperation, the maximum and median serum creatinine levels were 433.40 mmol/L and 81.60 mmol/L, respectively. By postoperative day 7, the corresponding values were 593.4 mmol/L and 83.3 mmol/L. RRT was administered to 25 AKI patients, with 12 achieving renal function recovery within 3 months. Among all AKI cases, 178 patients (88.6%) eventually recovered renal function. This group included 166 patients who recovered either spontaneously (particularly those with mild AKI) or following conservative medical management such as fluid resuscitation and terlipressin. Nevertheless, 15 patients exhibited persistent renal impairment that progressed to chronic kidney disease.

### Predictors of Early AKI Within 48 h Following LT

Univariate logistic regression analysis identified 16 significant predictors for the early-stage AKI (*P* < 0.05, Table 2). Collinearity diagnostics confirmed no severe collinearity among variables (Supplementary Table 4). Accordingly, all 16 significant variables from Table 2 were included in the initial multivariate adjustment. These predictors underwent backward stepwise selection in the multivariate logistic regression model, with rigorously predefined entry and removal thresholds of *P* < 0.05 and *P* > 0.10, respectively. Five independent predictors were identified and incorporated into the final model, including preoperative alcohol-associated cirrhosis (*P* = 0.021, odds ratio (OR) = 2.653), ALBI score ≥ -1.78 (*P* = 0.022, OR = 1.932), operation time ≥560 min (*P* < 0.001, OR = 3.017), intraoperative FFP transfusion (*P* = 0.010, OR = 2.255 per 1000 mL), and preoperative HE (*P* = 0.078, OR = 2.188) (Table 3).

**Table 2:** Univariable Logistic Regression Analysis of Predictors for Acute Kidney Injury (AKI) Within the First 48 h Following Liver Transplantation.

Candidate predictors	OR	95% CI	*P* value
Preoperative predictors			
Age, years	0.98	0.96–1.00	0.116
Male	0.85	0.48–1.51	0.578
BMI ≥28 kg/m^2^	2.21	1.14–4.26	0.018∗
MELD score ≥14	3.12	1.90–5.13	<0.001∗∗
Child–Pugh score ≥7	2.81	1.60–4.92	<0.001∗∗
ALBI score ≥ −1.78	2.94	1.78–4.85	<0.001∗∗
PNI <43	0.49	0.27–0.90	0.020∗
Diabetes mellitus	1.08	0.61–1.92	0.794
Hypertension	0.59	0.31–1.12	0.109
HE	3.41	1.61–7.19	0.001∗∗
Pathogenesis of liver disease			
HBV hepatitis	0.73	0.44–1.20	0.209
HCV hepatitis	0.98	0.29–3.29	0.973
Hepatocellular carcinoma	0.57	0.31–1.04	0.066
Alcohol-associated cirrhosis	2.64	1.23–5.69	0.013∗
Primary biliary cirrhosis	0.78	0.21–2.82	0.701
Pre-transplant BUN (mmol/L)	1.03	0.99–1.08	0.164
Pre-transplant SCr (mmol/L)	1.00	0.99–1.00	0.803
Intraoperative predictors			
Duration of hypotension ≥20 min	1.83	1.13–2.97	0.014∗
Operation time ≥560 min	3.64	2.20–6.01	<0.001∗∗
Cold ischemic time ≥400 min	2.06	1.22–3.45	0.007∗∗
Anhepatic phase ≥60 min	1.81	1.10–3.00	0.020∗
5% Albumin infusion ≥2000 mL	2.18	1.32–3.58	0.002∗∗
PRBCs’ infusion, units	1.10	1.04–1.17	0.001∗∗
FFP infusion, per 1000 mL	3.79	2.06–6.97	<0.001∗∗
Blood loss ≥1000 mL	2.98	1.66–5.35	<0.001∗∗
Urine output <1000 mL	0.58	0.34–0.97	0.039∗
Serum lactic acid maximum, mmol/L	1.08	0.98–1.20	0.128

Abbreviations: ALBI, albumin–bilirubin; BMI, body mass index; BUN, blood urea nitrogen; FFP, fresh frozen plasma; HE, hepatic encephalopathy; MELD, model for end-Stage liver disease; PNI, prognostic nutritional index; PRBCs, packed red blood cells; SCr, Serum Creatinine.

**Table 3:** Final Prediction Model for Acute Kidney Injury Within the First 48 h Following Liver Transplantation, Developed Using Multivariable Logistic Regression.

Variable	Regression coefficient	OR	95% CI	*P* value
Hepatic encephalopathy	0.751	2.118	0.920–4.874	0.078
Alcohol-related cirrhosis	0.976	2.653	1.156–6.089	0.021
ALBI score ≥ −1.78	0.658	1.932	1.100–3.393	0.022
Operation time ≥560 min	1.104	3.017	1.739–5.235	0.000
FFP infusion, per 1000 mL	0.813	2.25	1.218–4.175	0.010
Constant	−1.570	0.208		0.000

Abbreviations: ALBI, albumin–bilirubin; FFP, fresh frozen plasma; OR, odds ratio; CI, confidence interval.

### Model Results

The final results of the multivariate regression model are presented in Table 3.

The predictive model was expressed as follows: logit (P) = −1.570 + (0.751 × preoperative HE) + (0.976 × preoperative alcohol-associated cirrhosis) + (0.658 × ALBI score ≥ −1.78) + (1.104 × operation time ≥560 min) + (0.813 × intraoperative FFP transfusion per 1000 mL).

**Figure 2:**
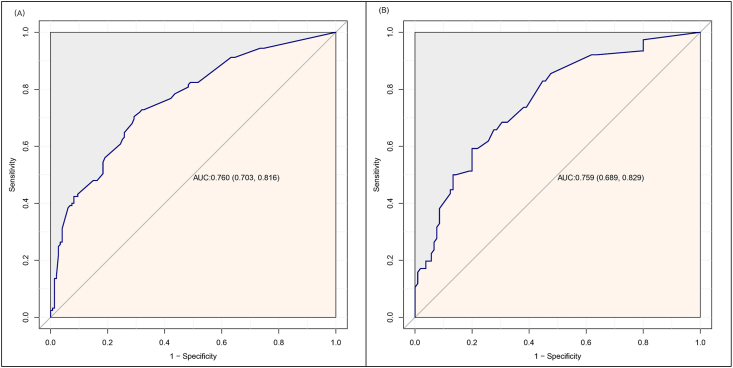
ROC curves of the model predicting the occurrence of 48h AKI after liver transplantation (LT). The area under the curve (AUC) was 0.760 (95% CI: 0.703–0.816) in the development cohort (A) and 0.759 (95% CI: 0.689–0.829) in the validation cohort (B). AKI, acute kidney injury; CI, confidence interval; LT, liver transplantation.

**Figure 3:**
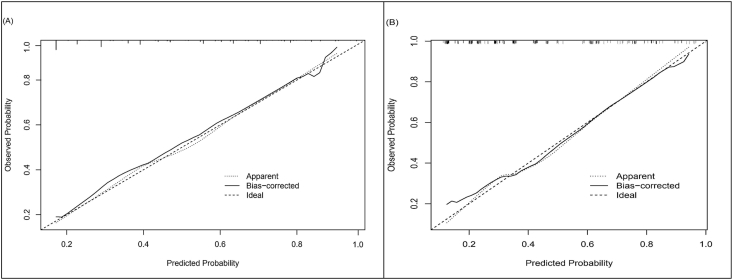
Calibration plots comparing predicted and observed probabilities of 48h AKI after liver transplantation (LT) in the development cohort (A) and the validation cohort (B).

**Figure 4:**
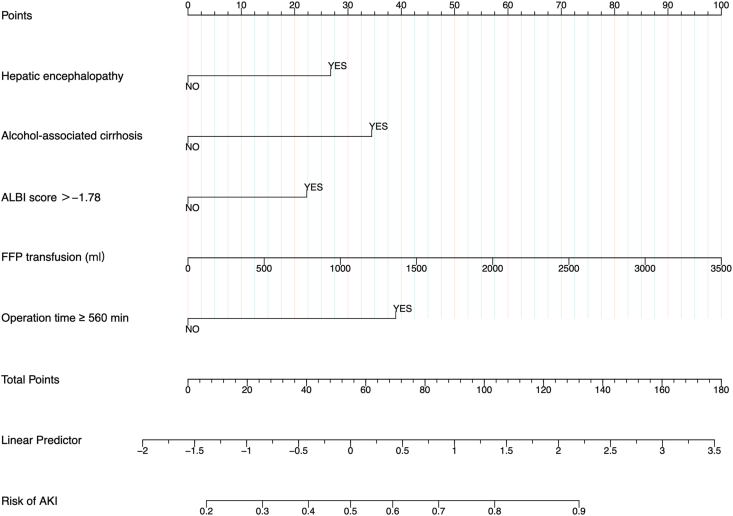
Nomogram for predicting early AKI within 48 h after LT. To use, draw a vertical line from each variable to the ‘Points’ axis to assign a score. Sum the scores and locate the total on the ‘Total Points’ axis. Finally, draw a vertical line to the ‘Risk of AKI’ axis to determine the predicted probability of AKI. Abbreviations: AKI, acute kidney injury; FFP, fresh frozen plasma; LT, liver transplantation.

The model, incorporating these five predictors, demonstrated strong discrimination. In the development cohort, the AUROC was 0.760 (95% CI: 0.703–0.816, Figure 2A), and the Hosmer–Lemeshow test yielded a Chi-square value of 6.8 (degrees of freedom [d.f.] = 8, *P* = 0.555). In the validation cohort, the AUROC was 0.759 (95% CI: 0.689–0.829, Figure 2B), and the Hosmer–Lemeshow test yielded a Chi-square value of 9.0 (d.f. = 7, *P* = 0.253). The diagnostic performance metrics of the model were as follows: sensitivity of 0.592, specificity of 0.867, negative predictive value of 0.746, and positive predictive value of 0.763. Calibration curves demonstrated near-linear agreement between predicted and actual risks, with slight underestimation at extreme probabilities (Figure 3B). A nomogram (Figure 4) was developed to visually represent the contribution of each predictor, facilitating clinical application.

### Risk Factors for Early Severe AKI Following LT

Univariate and multivariate logistic regression analyses identified independent risk factors for severe AKI, including operation time ≥560 min, MELD score ≥14, intraoperative blood loss ≥1000 mL, urine output <1000 mL, and elevated intraoperative lactate level (Table 4). These findings underscore the importance of optimizing perioperative management to mitigate AKI risk.

**Table 4:** Univariable and Multivariable Logistic Regression Analyses of Risk Factors for Severe Acute Kidney Injury (AKI) Within the First 48 h After Liver Transplantation.

	Univariate logistic regression		Multivariate regression	
Variables	OR 95% CI	*P* value	OR 95% CI	*P* value
Preoperative Variables				
Age, year	1.00 (0.97–1.02)	0.746		
Female	0.68 (0.39–1.19)	0.177		
BMI ≥28 kg/m^2^	1.74 (0.93–3.25)	0.083		
Diabetes mellitus	0.72 (0.38–1.38)	0.324		
Hypertension	0.59 (0.28–1.24)	0.163		
HE	2.33 (1.29–4.21)	0.005		
MELD score ≥14	2.71 (1.58–4.67)	<0.001	2.10 (1.18–3.73)	0.012
ALBI score > −1.78	1.93 (1.14–3.27)	0.014		
Child–Pugh score ≥8.5	2.19 (1.32–3.65)	0.003		
PNI >42	0.81 (0.46–1.44)	0.474		
Pathogenesis of liver disease				
HBV hhepatitis	0.85 (0.51–1.4)	0.513		
HCV hhepatitis	0.55 (0.12–2.41)	0.426		
Hepatocellular carcinoma	0.85 (0.45–1.61)	0.626		
Alcohol-related cirrhosis	1.34 (0.66–2.74)	0.423		
Primary biliary cirrhosis	1.20 (0.39–3.66)	0.753		
Pre-transplant BUN (mmol/L)	1.01 (0.96–1.06)	0.715		
Pre-transplant SCr (mmol/L)	0.99 (0.98–1)	0.082		
Intraoperative Variables				
Severe Hypotension	1.83 (1.09–3.06)	0.022		
Cold ischemic time ≥340 min	1.42 (0.84–2.42)	0.194		
Operation time ≥560 min	2.73 (1.63–4.55)	<0.001	1.90 (1.08–3.34)	0.025
Anhepatic phase ≥60 min	1.81 (1.05–3.11)	0.033		
Duration of hypotension ≥10 min	2.00 (1.08–3.72)	0.028		
5% AAlbumin infusion ≥2000 mL	1.66 (0.98–2.82)	0.058		
PRBC_S_ infusion, units	1.11 (1.06–1.17)	<0.001		
FFP infusion, per 1000 mL	2.11 (1.37–3.26)	0.001		
Blood loss ≥1000 mL	3.40 (2.01–5.74)	<0.001	2.39 (1.34–4.28)	0.003
Urine output <1000 mL	0.48 (0.29–0.81)	0.005	2.13 (1.23–3.71)	0.007
Serum lactic acid maximum, mmol/L	1.15 (1.05–1.26)	0.004	1.09 (1.01–1.18)	0.038

Abbreviations: ALBI, albumin–bilirubin; BMI, body mass index; BUN, blood urea nitrogen; FFP, fresh frozen plasma; HE, hepatic encephalopathy; MELD, model for end-Stage liver disease; PNI, prognostic nutritional index; PRBCs, packed red blood cells; SCr, Serum Creatinine.

## DISCUSSION

Early detection of AKI within the first 48 h following LT is vital for preventing long-term renal dysfunction and improving outcomes. This critical period, during which renal injury remains highly reversible, presents a valuable opportunity for targeted interventions to improve recovery trajectories. The proposed predictive model could specifically target AKI within this timeframe, emphasizing the importance of timely recognition. Key predictors included preoperative HE, alcohol-associated cirrhosis, ALBI score ≥ -1.78, operation time ≥560 min, and intraoperative FFP transfusion. Identifying high-risk patients during this 48-h time window enables early interventions, such as optimizing hemodynamics and minimizing nephrotoxic exposures, which can significantly reduce severe AKI risk, improve renal function, and enhance posttransplant recovery.

Severe AKI, which is closely linked to higher risks of early RRT, CKD, heart failure, and mortality, was reported to be strongly associated with several key preoperative and intraoperative factors.^12^ In addition to the prolonged operation time, a higher MELD score, substantial blood loss, urine output <1000 mL, and the elevated lactate level were identified as independent risk factors for severe AKI. These findings further highlight the importance of proactive perioperative management, particularly during the first 48 h following transplantation. Early identification and intervention of high-risk patients can significantly alter recovery trajectories and reduce the risk of long-term complications. Thus, the proposed predictive model could provide valuable insights into preventing severe AKI and improving overall patient prognosis following LT.

Among the preoperative factors analyzed in this study, HE was examined due to its relationship with AKI in patients with advanced liver disease. The severity of liver disease is widely recognized as a key driver of AKI.^29^ HE, a severe complication of decompensated cirrhosis, is a complex but potentially reversible neuropsychiatric syndrome that is closely associated with patient prognosis.^30^^,^^31^ Previous research demonstrated that the prevalence of HE in cirrhotic patients ranges from 35% to 45%.^30^ Its diagnosis relies primarily on clinical manifestations, such as asterixis, psychomotor slowing,^32^ extrapyramidal symptoms,^33^ somnolence, seizures,^34^ and coma,^35^ mainly supported by ancillary tests. Epidemiological data further highlighted the strong association between HE and AKI. A meta-analysis by Lekakis *et al.* reported an AKI incidence of up to 41% among cirrhotic patients with HE.^29^ Moreover, a study by Catalán *et al.* demonstrated that preoperative HE significantly increased the risk of postoperative AKI in liver transplant recipients, particularly severe AKI.^36^ These findings suggest that HE not only indicates severe liver dysfunction but may also exacerbate renal impairment through mechanisms, including systemic inflammation and hemodynamic instability. Notably, the present study did not identify HE as an independent risk factor for AKI within the first 48 h following LT. This finding may be attributed to the relatively low prevalence of HE in the study cohort, as well as inconsistencies in HE grading, treatment approaches, and intraoperative management strategies. Despite these limitations, HE remains a critical complication of cirrhosis, and its potential role in the pathogenesis of AKI following LT requires further exploration and validation.

In this study, prolonged operation time (≥560 min) was identified as the most significant predictive factor for postoperative outcomes. Extended operation time serves as a comprehensive indicator, not only indicating the prolonged duration of various surgical phases, such as the pre-anhepatic, anhepatic, and neohepatic phases, but also the increased complexity of the underlying disease and the technical challenges associated with the procedure.^16^ The analysis demonstrated that when the operation time exceeded 560 min, the risk of early postoperative AKI significantly increased, with a threefold elevation in risk (OR = 3.017, *P* < 0.001). This finding is consistent with previous research, which has shown a higher incidence of AKI in orthotopic LT when operation time surpassed 480 min, substantially raising the AKI risk.^37^ Additionally, retrospective analysis of large patient cohorts has revealed that prolonged operation time not only extends intensive care unit stay but also serves as an independent risk factor for AKI in adult living donor LT.^38^ In various predictive models for AKI following LT, operation time consistently proves to be a critical and reliable determinant,^14^^,^^37^^,^^38^ emphasizing its crucial role in risk stratification.

This study identified alcohol-associated cirrhosis as the second most significant predictive factor for postoperative AKI. Alcohol-associated cirrhosis has been consistently recognized as an independent risk factor for the increased AKI risk following LT.^39^ As a common cause of end-stage liver disease, advanced cirrhosis is associated with a hyperdynamic circulatory state, including increased cardiac output and reduced systemic vascular resistance. These circulatory abnormalities, particularly in patients with ascites, lead to reduced effective circulating blood volume, reflexive renal sympathetic activation, severe renal vasoconstriction, and ultimately hepatorenal syndrome and renal failure.^37^

The findings of the present study revealed that preoperative alcohol-associated cirrhosis increased the risk of early postoperative AKI by 2.65-fold (OR = 2.65, *P* = 0.021). This aligns with prior studies demonstrating a significantly elevated AKI risk in patients with alcoholic hepatitis and a strong association between preoperative alcoholic liver disease and AKI following LT.^10^^,^^38^ Although alcoholic liver disease has been found to be linked to late-stage renal dysfunction posttransplant, its specific impact on early postoperative renal outcomes warrants further investigation.^40^

The heightened AKI risk in alcohol-associated cirrhosis may stem from alcohol’s effects on the hepatic function and systemic inflammatory responses. Newey *et al.* identified alcohol as the most frequent etiology of hepatic encephalopathy,^34^ indirectly supporting the notion that patients with alcohol-associated cirrhosis are more prone to postoperative AKI. Similarly, Michelena *et al.* demonstrated that systemic inflammatory response syndrome (SIRS) is a major mechanism underlying multiple organ failure in alcoholic liver disease, and renal failure accounts for 34%.^41^ On the other hand, other scholars confirmed that AKI could significantly increase the risk of complications in alcohol-associated cirrhosis,^42^ including hepatorenal syndrome, HE, and mortality.^43^ These findings collectively highlight the complex interaction among alcohol-induced liver dysfunction, systemic inflammation, and renal impairment, emphasizing the need for targeted strategies to mitigate AKI risk in this population.

The present study identified a preoperative ALBI score of ≥ −1.78 as an independent risk factor for early postoperative AKI in LT patients. First introduced in 2015, the ALBI score was designed for objectively assessing hepatic function and predicting survival in patients with hepatocellular carcinoma. ^28^ In contrast to traditional scoring systems, such as the Child–Pugh classification and the MELD score, the ALBI score relies solely on serum albumin and bilirubin levels, avoiding subjective factors, such as ascites and HE, thereby ensuring greater accuracy and reproducibility. The ALBI scoring has since been validated and widely applied in various liver diseases, including primary biliary cirrhosis^44^ and HBV-related acute-on-chronic liver failure.^45^ It has demonstrated superiority over the MELD score in predicting outcomes following LT.^46^^,^^47^ Higher preoperative ALBI scores have been consistently associated with the increased risk of postoperative AKI,^46^ highlighting its prognostic value. Furthermore, the ALBI score is clinically practical as it requires only routine blood test parameters.^44^ Serum albumin, a key component of the ALBI score, plays a remarkable role in AKI risk assessment, in which every 10 g/L decrease is associated with a 1.34-fold increase in AKI risk^48^ and a 1.47-fold increase in mortality risk.^49^,^50^ Elevated bilirubin level, indicative of liver injury or biliary obstruction, further enhances the ALBI score’s utility in predicting AKI and other postoperative complications.

This study also revealed that the volume of intraoperative FFP transfusion was independently associated with the development of postoperative AKI, aligning with findings of prior research.^51^,^52^ FFP, rich in coagulation factors and antithrombin, is a critical intervention for managing intraoperative bleeding and coagulopathy during LT.^48^ Coagulation dysfunction and hyperfibrinolysis may partially reflect severe IRI and early graft dysfunction. In the present study, each additional 1000 mL of intraoperative FFP transfusion was associated with a 2.2-fold increase in the risk of postoperative AKI.

Intraoperative transfusion of large volumes of blood products may reflect poor preoperative baseline conditions, substantial intraoperative blood loss, and coagulation abnormalities. However, transfusions can also exacerbate IRI,^53^ directly impairing the function of distal organs, such as the kidneys, lungs, and intestines. This effect is compounded by the hemodynamic instability^54^ that increases susceptibility to AKI. Moreover, IRI can worsen SIRS, further elevating the risk of postoperative AKI.^11^ To address these risks, comprehensive preoperative optimization, advancements in surgical techniques, reduction of operation time, and developing strategies to minimize intraoperative bleeding and transfusion volumes are essential. These measures are pivotal for reducing the incidence of AKI and enhancing postoperative outcomes in LT.

This study examined risk factors for early postoperative AKI, particularly within 48 h, following LT. In contrast to prior research, factors such as BMI, hypertension, diabetes, MELD score, Child–Pugh score, and blood loss were not detected as independent predictors, potentially owing to differences in study endpoints and population characteristics. Notably, albumin and bilirubin levels, key components of the MELD and Child–Pugh scores, were integrated into the final predictive model via the ALBI score, providing a novel and more precise indicator. Furthermore, blood loss may indirectly contribute to AKI risk by exacerbating coagulopathy as indicated by the volume of FFP transfused.

### Strengths and Limitations

This study demonstrated several notable strengths. Firstly, it relied on a large, high-quality dataset with minimal missing values, which derived from a single organ transplantation center that employed rigorous and standardized clinical definitions. The development and validation cohorts were randomly sampled, exhibiting no significant differences, and the model was strongly validated. Secondly, AKI was defined using the 2012 KDIGO criteria, which provided superior prognostic accuracy compared with alternative definitions. Thirdly, the model utilized readily accessible perioperative variables, involving a broad spectrum of predictive factors, and complied with the TRIPOD guidelines. Importantly, it is applicable immediately following surgery, enabling clinicians to promptly identify high-risk patients and implement early interventions.

However, the study has certain limitations. As a retrospective, single-center analysis, it may not fully address potential confounders. Ascites was not adjusted for due to nonsignificant association with BMI. Donor-related data were unavailable due to privacy restrictions. Comprehensive postoperative infection data were not collected due to the study’s focus on AKI-specific predictors. Additionally, emerging postoperative factors, such as delirium, which have been increasingly associated with early AKI risk, were not included due to their later onset and the low proportion of elderly patients in the cohort. Furthermore, while the KDIGO definition used in this study relied solely on the serum creatinine level without incorporating urine output, this limitation was likely minimal given the variability of urine output influenced by fluid status and diuretics. Finally, external validation through high-quality, multicenter, prospective cohorts was necessary to enhance the model’s generalizability and optimize its clinical applicability.

A predictive model was developed and visualized as a nomogram to quantify the risk of early AKI within 48 h following LT. Using preoperative and intraoperative predictors, the model demonstrated strong discriminatory ability and calibration in internal validation. It could enable clinicians to assess postoperative AKI risk immediately after surgery, providing timely risk stratification and guiding early interventions for high-risk patients. The findings highlighted the importance of optimizing preoperative conditions, such as treating HE and improving liver function, to reduce AKI incidence and improve patient outcomes.

## Data availability

The data underlying this article will be shared on reasonable request to the corresponding author.

## Ethical compliance

This study was approved by the Institutional Review Board of the Beijing Tsinghua Changgung Hospital, School of Clinical Medicine, Tsinghua University (protocol No.22216-4-01). Written informed consent was not needed from the patients’ parents or legal guardians because of the retrospective design of this study and the anonymous collection of analytical data.

## Credit authorship contribution statement

Yuzhi Wei, Ziheng Qi, and Huan Zhang contributed to conception and design.

Wenyan Wu, Haolin Yin, Chunyu Feng, Bo Yang, Caiyun Zhang, Xiaoyan Gao, Haotian Wu, Shichao Sun contributed to collection and assembly of data.

Wenfang Zhang, Yuzhi Wei, Ziheng Qi, and Huan Zhang contributed to data analysis and interpretation.

All authors contributed to manuscript writing.

All authors contributed to final approval of manuscript.

## Clinical trial registration

The trial was registered on the ClinicalTrials.gov registry (Identifier: NCT06750770).

URL: https://clinicaltrials.gov/study/NCT06750770?term=NCT06750770&rank=1

## Funding

This work was supported by the Strategic Projects for Accurate Medicine Science Research Program, Tsinghua University (Grant No. 2022ZLB009).

## Declaration of competing interest

The authors declare the following financial interests/personal relationships which may be considered as potential competing interests: Huan Zhang reports financial support was provided by the Strategic Projects for Accurate Medicine Science Research Program, Tsinghua University. If there are other authors, they declare that they have no known competing financial interests or personal relationships that could have appeared to influence the work reported in this paper.
