# Supplementary material for: Early Prediction of Acute Kidney Injury Following Liver Transplantation: Development and Validation of a Clinical Risk Model
Source: J Clin Exp Hepatol. 2025 Aug 29;16(1):103179. doi: 10.1016/j.jceh.2025.103179 (PMC12493209; doi:10.1016/j.jceh.2025.103179)
Supplement: Multimedia component 1 [file mmc1.docx]

Supplementary table 1 The initial diagnosis and staging of acute kidney injury according to 2012 KDIGO

| Stage | Serum creatinine | Urine output |
| --- | --- | --- |
| 1 | 1.5–1.9 times baseline  or  ≥0.3 mg/dl (≥26.5 μmol/l) increase | <0.5 ml/kg/h for 6–12 h |
| 2 | 2.0–2.9 times baseline | <0.5 ml/kg/h for ≥12 h |
| 3 | 3 times baseline  or  ≥4.0 mg/dl (≥353.6 μmol/l) increase  or  initiation of RRT  or  in patients <18 years a decrease in eGFR  <35 ml/min/1.73 m^2^ | <0.3 ml/kg/h for ≥24 h  or  anuria ≥12 h |

Abbreviations: KDIGO , kidney disease improving global outcomes; RRT, renal replacement therapy；eGFR: estimated glomerular filtration rate.
