# Supplementary material for: Early Prediction of Acute Kidney Injury Following Liver Transplantation: Development and Validation of a Clinical Risk Model
Source: J Clin Exp Hepatol. 2025 Aug 29;16(1):103179. doi: 10.1016/j.jceh.2025.103179 (PMC12493209; doi:10.1016/j.jceh.2025.103179)
Supplement: Multimedia component 2 [file mmc2.docx]

**Supplementary table 2. Selection of cut-off values for continuous variables**

| **Variable ROC Item** | **Value** | **Final value** |
| --- | --- | --- |
| **MELD score threshold** | **13.5** | **14** |
| MELD score sensitivity | 0.664 |  |
| MELD score specificity | 0.6122 |  |
| MELD score ppv | 0.5929 |  |
| MELD score npv | 0.6818 |  |
| MELD score accuracy | 0.636 |  |
| MELD score precision | 0.5929 |  |
| **ALBI score threshold** | **-1.775** | **-1.78** |
| ALBI score sensitivity | 0.688 |  |
| ALBI score specificity | 0.5714 |  |
| ALBI score ppv | 0.5772 |  |
| ALBI score npv | 0.6829 |  |
| ALBI score accuracy | 0.625 |  |
| ALBI score precision | 0.5772 |  |
| **Child-Pugh score threshold** | **7.5** | **7** |
| Child-Pugh score sensitivity | 0.736 |  |
| Child-Pugh score specificity | 0.4966 |  |
| Child-Pugh score ppv | 0.5542 |  |
| Child-Pugh score npv | 0.6887 |  |
| Child-Pugh score accuracy | 0.6066 |  |
| Child-Pugh score precision | 0.5542 |  |
| **PNI threshold** | **43.425** | **43** |
| PNI sensitivity | 0.872 |  |
| PNI specificity | 0.2721 |  |
| PNI ppv | 0.5046 |  |
| PNI npv | 0.7143 |  |
| PNI accuracy | 0.5478 |  |
| PNI precision | 0.5046 |  |
| **Cold ischemia time threshold** | **394.5** | **400** |
| Cold ischemia time sensitivity | 0.424 |  |
| Cold ischemia time specificity | 0.7347 |  |
| Cold ischemia time ppv | 0.5761 |  |
| Cold ischemia time npv | 0.6 |  |
| Cold ischemia time accuracy | 0.5919 |  |
| Cold ischemia time precision | 0.5761 |  |
| **Operation time threshold** | **558.5** | **560** |
| Operation time sensitivity | 0.624 |  |
| Operation time specificity | 0.6939 |  |
| Operation time ppv | 0.6341 |  |
| Operation time npv | 0.6846 |  |
| Operation time accuracy | 0.6618 |  |
| Operation time precision | 0.6341 |  |
| **Anhepatic phase threshold** | **69** | **60** |
| Anhepatic phase sensitivity | 0.536 |  |
| Anhepatic phase specificity | 0.6531 |  |
| Anhepatic phase ppv | 0.5678 |  |
| Anhepatic phase npv | 0.6234 |  |
| Anhepatic phase accuracy | 0.5993 |  |
| Anhepatic phase precision | 0.5678 |  |
| **Duration of hypotension threshold** | **22.5** | **20** |
| Duration of hypotension sensitivity | 0.544 |  |
| Duration of hypotension specificity | 0.6395 |  |
| Duration of hypotension ppv | 0.562 |  |
| Duration of hypotension npv | 0.6225 |  |
| Duration of hypotension accuracy | 0.5956 |  |
| Duration of hypotension precision | 0.562 |  |
| **5% Albumin infusion threshold** | **2025** | **2000** |
| 5% Albumin infusion sensitivity | 0.672 |  |
| 5% Albumin infusion specificity | 0.517 |  |
| 5% Albumin infusion ppv | 0.5419 |  |
| 5% Albumin infusion npv | 0.6496 |  |
| 5% Albumin infusion accuracy | 0.5882 |  |
| 5% Albumin infusion precision | 0.5419 |  |
| **Urine output threshold** | **895** | **1000** |
| Urine output sensitivity | 0.344 |  |
| Urine output specificity | 0.8367 |  |
| Urine output ppv | 0.6418 |  |
| Urine output npv | 0.6 |  |
| Urine output accuracy | 0.6103 |  |
| Urine output precision | 0.6418 |  |

Abbreviations: PPV: Positive Predictive Value; NPV, Negative Predictive Value
