# Supplementary material for: Early Prediction of Acute Kidney Injury Following Liver Transplantation: Development and Validation of a Clinical Risk Model
Source: J Clin Exp Hepatol. 2025 Aug 29;16(1):103179. doi: 10.1016/j.jceh.2025.103179 (PMC12493209; doi:10.1016/j.jceh.2025.103179)
Supplement: Multimedia component 3 [file mmc3.docx]

Supplementary table 3. Perioperative characteristics of patients between development and validation cohort.

| Predictors | Total | Development cohort | Validation cohort | *P* value |
| --- | --- | --- | --- | --- |
|  | (n = 453) | (n = 272) | (n = 181) |  |
| Preoperative predictors |  |  |  |  |
| Age, median (IQR), year | 52.0 (45.0, 60.0) | 52.0 (44.0, 60.0) | 52.0 (46.0, 60.0) | 0.814 |
| Female, n (%) | 100 (22.1) | 59 (21.7) | 41 (22.7) | 0.809 |
| BMI ≥ 28kg/m^2^, n (%) | 67 (14.8) | 45 (16.5) | 22 (12.2) | 0.197 |
| Diabetes mellitus, n (%) | 98 (21.6) | 59 (21.7) | 39 (21.5) | 0.971 |
| Hypertension, n (%) | 80 (17.7) | 48 (17.6) | 32 (17.7) | 0.993 |
| HE, n (%) | 71 (15.7) | 38 (14.0) | 33 (18.2) | 0.222 |
| Pathogenesis of liver disease, n (%) |  |  |  |  |
| HBV hepatitis | 275 (60.7) | 174 (64.0) | 101 (55.8) | 0.081 |
| HCV hepatitis | 20 (4.4) | 11 (4.0) | 9 (5.0) | 0.638 |
| Hepatocellular carcinoma | 94 (20.8) | 57 (21.0) | 37 (20.4) | 0.895 |
| Alcohol-related cirrhosis | 54 (11.9) | 33 (12.1) | 21 (11.6) | 0.865 |
| Primary biliary cirrhosis | 21 (4.6) | 10 (3.7) | 11 (6.1) | 0.234 |
| MELD score ≥ 14, n (%) | 238 (52.5) | 140 (51.5) | 98 (54.1) | 0.577 |
| Child-Pugh score ≥ 7, n (%) | 324 (71.5) | 192 (70.6) | 132 (72.9) | 0.589 |
| ALBI score ≥ -1.78, n (%) | 204 (45.0) | 122 (44.9) | 82 (45.3) | 0.925 |
| PNI＜43,n (%) | 347 (76.6) | 211 (77.6) | 136 (75.1) | 0.549 |
| Pre-transplant BUN, median (IQR), mmol/L | 5.1 (3.9, 7.1) | 5.2 (3.9, 7.1) | 5.1 (3.9, 7.0) | 0.970 |
| Pre-transplant SCr, median (IQR), mmol/L | 61.0 (49.8, 75.5) | 59.4 (49.9, 76.2) | 63.0 (49.8, 75.2) | 0.818 |
| Intraoperative predictors |  |  |  |  |
| Operation time ≥ 560 min, n (%) | 191 (42.2) | 122 (44.9) | 69 (38.1) | 0.155 |
| Blood loss ≥ 1000 ml, n (%) | 104 (23.0) | 65 (23.9) | 39 (21.5) | 0.560 |
| Urine output＜1000 ml, n (%) | 132 (29.1) | 83 (30.5) | 49 (27.1) | 0.430 |
| Cold ischemia time≥ 400 min, n (%) | 142 (31.3) | 86 (31.6) | 56 (30.9) | 0.879 |
| Anhepatic phase ≥60 min, n (%) | 276 (60.9) | 169 (62.1) | 107 (59.1) | 0.519 |
| 5% Albumin infusion ≥2000 ml, n (%) | 263 (58.1) | 160 (58.8) | 103 (56.9) | 0.685 |
| Duration of hypotension ≥ 20min, n (%) | 231 (51.0) | 139 (51.1) | 92 (50.8) | 0.954 |
| PRBC_S_ infusion, median (IQR), units | 4.0 (0.0, 8.0) | 4.0 (0.0, 8.0) | 4.0 (0.0, 8.0) | 0.498 |
| FFP infusion, median (IQR), ml | 400.0 (0.0, 400.0) | 400.0 (0.0, 450.0) | 400.0 (0.0, 400.0) | 0.620 |
| Serum lactic acid maximum, median (IQR), mmol /L | 3.6 (2.8, 4.8) | 3.6 (2.7, 4.6) | 3.6 (2.9, 5.1) | 0.184 |

Abbreviations: BMI, body mass index; HE, hepatic encephalopathy; MELD, model for end-Stage liver disease; ALBI, albumin-bilirubin; PNI, prognostic nutritional index; BUN, blood urea nitrogen; SCr, Serum Creatinine; PRBCs, packed red blood cells; FFP, fresh frozen plasma.
