# Supplementary material for: Early Prediction of Acute Kidney Injury Following Liver Transplantation: Development and Validation of a Clinical Risk Model
Source: J Clin Exp Hepatol. 2025 Aug 29;16(1):103179. doi: 10.1016/j.jceh.2025.103179 (PMC12493209; doi:10.1016/j.jceh.2025.103179)
Supplement: Multimedia component 4 [file mmc4.docx]

Supplementary table 4. Covariance diagnosis of predictors.

| Model | | Unnormalized coefficient | | | Standardization coefficient |  |  | Collinearity statistics | |
| --- | --- | --- | --- | --- | --- | --- | --- | --- | --- |
|  |  | B | | Standard error | Beta | t | *P* value | Tolerance | VIF |
| 1 | Constant | | -0.389 | 0.267 |  | -1.455 | 0.147 |  |  |
|  | BMI ≥ 28kg/m^2^ | | 0.035 | 0.078 | 0.026 | 0.446 | 0.656 | 0.881 | 1.134 |
|  | HE | | 0.147 | 0.087 | 0.103 | 1.699 | 0.091 | 0.825 | 1.212 |
|  | MELD Score ≥ 14 | | 0.112 | 0.081 | 0.112 | 1.378 | 0.170 | 0.453 | 2.207 |
|  | Child Pugh Score ≥ 7 | | -0.048 | 0.090 | -0.047 | -.534 | 0.594 | 0.390 | 2.567 |
|  | ALBI^*^ Score ≥ -1.78 | | 0.111 | 0.085 | 0.111 | 1.297 | 0.196 | 0.413 | 2.421 |
|  | PNI^#^＜43 | | 0.001 | 0.077 | 0.001 | 0.017 | 0.986 | 0.715 | 1.399 |
|  | Alcohol-related cirrhosis | | 0.182 | 0.090 | 0.119 | 2.034 | 0.043 | 0.874 | 1.144 |
|  | Duration of hypotension ≥ 20 min | | 0.016 | 0.060 | 0.016 | 0.264 | 0.792 | 0.843 | 1.186 |
|  | Operation time ≥ 560 min | | 0.223 | 0.079 | 0.223 | 2.813 | 0.005 | 0.480 | 2.082 |
|  | Cold ischemic time≥ 400 min | | -0.021 | 0.082 | -0.020 | -.262 | 0.793 | 0.518 | 1.932 |
|  | Anhepatic phase ≥ 60 min | | 0.084 | 0.059 | 0.082 | 1.434 | 0.153 | 0.915 | 1.092 |
|  | 5% Albumin infusion≥ 2000 ml | | 2.348E-5 | 0.000 | 0.054 | 0.794 | 0.428 | 0.647 | 1.546 |
|  | PRBCs^‡^ infusion, units | | -0.015 | 0.011 | -0.149 | -1.422 | 0.156 | 0.275 | 3.631 |
|  | FFP infusion, per 1000ml | | 0.000 | 0.000 | 0.163 | 1.699 | 0.090 | 0.328 | 3.046 |
|  | Urine output <1000 ml | | -0.108 | 0.064 | -0.100 | -1.686 | 0.093 | 0.853 | 1.172 |
|  | Blood loss≥1000 ml | | 0.138 | 0.088 | 0.118 | 1.568 | 0.118 | 0.528 | 1.893 |

Abbreviations: BMI, body mass index; HE, hepatic encephalopathy; MELD score, Model for End-Stage Liver Disease; ALBI^*^ score, albumin-bilirubin score; PNI^#^, prognostic nutritional index; PRBCs^‡^, packed red blood cells; FFP, fresh frozen plasma.
